# From One Ejaculate to Another: Transference of Sperm Traits via Seminal Plasma Supplementation in the Ram

**DOI:** 10.3390/biology9020033

**Published:** 2020-02-18

**Authors:** Christine Green, Jessica P. Rickard, Simon P. de Graaf, Angela J. Crean

**Affiliations:** 1 School of Life and Environmental Sciences, The University of Sydney, Sydney, NSW 2006, Australia; 2 Sydney School of Veterinary Science, The University of Sydney, Sydney, NSW 2006, Australia

**Keywords:** semen, inter-ejaculate variability, ram, assisted reproductive technologies, male fertility, sperm competition

## Abstract

Males can adjust sperm motility instantaneously in response to the perceived risk of sperm competition. The speed of this response suggests that sperm motility is regulated by changes in seminal plasma rather than changes in the sperm cells themselves. Hence, here we test whether inter-ejaculate variation in seminal plasma can be used to alter sperm quality prior to use in assisted reproductive technologies. We supplemented fresh ejaculates of Merino rams with seminal plasma collected from previous ‘donor’ ejaculates to test whether changes in sperm kinetics were related to the relative quality of donor to focal ejaculates. We found a positive relationship between the change in sperm traits before and after supplementation, and the difference in sperm traits between the donor and focal ejaculate. Hence, sperm motility can be either increased or decreased through the addition of seminal plasma from a superior or inferior ejaculate, respectively. This positive relationship held true even when seminal plasma was added from a previous ejaculate of the same ram, although the slope of the relationship depended on the identity of both the donor and receiver ram. These findings indicate that seminal plasma plays a key role in the control and regulation of sperm kinetics, and that sperm kinetic traits can be transferred from one ejaculate to another through seminal plasma supplementation.

## 1. Introduction

Males show a remarkable ability to facultatively adjust both the quantity and quality of sperm released in an ejaculate in response to the perceived risk that their sperm will have to compete against another male’s sperm to fertilise an egg [1]. In some cases, these changes in sperm motility can occur almost instantaneously. For example, in humans, males produce ejaculates with more motile sperm after viewing stimulus material with competitor males [2] and novel females [3]. A full spermatogenic cycle in humans takes over 70 days [4]. Even after sperm are released from the testis, sperm spend 5 to 6 days transiting through the epididymis, during which time the final steps of maturation are regulated by exogenous rather than genomic factors [5,6]. Hence, instantaneous changes in sperm motility are likely to be driven by changes in seminal plasma composition rather than changes in the sperm cells themselves.

In mammals, an ejaculate consists of spermatozoa in testicular and epididymal fluids mixed with secretions from the seminal vesicles, prostate and bulbourethral glands (collectively termed the accessory sex glands). While seminal plasma was originally considered to function exclusively as a transport medium for sperm, it is now recognised to play an important role in many aspects of male reproductive success by influencing factors including sperm physiology, male/female interactions, and even offspring health [7,8,9]. Seminal plasma remodels the sperm surface and modifies the physiological response of spermatozoa through the provision of signalling factors and glycoproteins with sperm-binding properties [10]. Its composition varies among species, breeds, males, and ejaculates (e.g., across seasons and ages) [11,12], suggesting that seminal plasma composition is responsive to changes in selective pressures and environmental conditions.

One context in which seminal plasma has received significant research attention is in the application of assisted reproductive technologies (ART). Epididymal sperm can be successfully used to fertilise eggs both in vitro and in vivo [13], demonstrating that seminal plasma is not essential for reproduction [14]. Indeed, seminal plasma is routinely washed from sperm prior to use in ART as its persistent presence in IVF prevents capacitation and sperm-egg fusion [15]. However, seminal plasma supplementation during sperm processing can also be used to improve sperm function in ART [10,13], although outcomes are inherently variable due to variability in seminal plasma composition and the method of supplementation [16]. For example, the addition of seminal plasma can improve post-thaw sperm quality, but only when the seminal plasma is sourced from males with high freeze-tolerance ejaculates [17,18]. In addition, the effects of self versus non-self seminal plasma supplementation in stallions depend on the origin of both the seminal plasma and the spermatozoa [19]. Hence, to fully understand the regulation of spermatozoa by seminal plasma, effects need to be examined at the level of the ejaculate.

Seminal plasma is often pooled from multiple ejaculates or multiple males before use as a supplement to control for effects of variation in seminal plasma composition e.g., [12,17,18]. However, variation at the level of the ejaculate could be exploited to enhance sperm traits without the biosecurity risk of supplementing sperm samples with seminal plasma from another male. For instance, poor quality ejaculates collected at the fringes or out of the breeding season could be at least partially rescued by supplementing with seminal plasma collected during the peak breeding season [12]. Or seminal plasma collected at the peak of a male’s condition could be used to enhance sperm performance later in life. To test whether (a) sperm function is regulated through inter-ejaculate changes in seminal plasma, and (b) inter-ejaculate seminal plasma supplementation can be used to shift sperm traits, we tested whether changes in sperm kinematic traits after seminal plasma supplementation could be predicted from the relative difference in sperm traits between donor and focal ejaculates. We used a fully crossed design, so that all focal samples were supplemented with both self and foreign seminal plasma samples.

## 2. Materials and Methods

### 2.1. General Methods

#### 2.1.1. Animals and Semen Collection

Ejaculates were collected from three, mature age (3–5 years old), Merino rams (Ram A, B and C) housed at the University of Sydney Camperdown campus, selected for their high libido and ejaculate quality. The same three rams were used as both donors and receivers in this study, as we were mainly interested in variation at the level of individual ejaculates. All ejaculates were collected via artificial vagina (June–July 2018), with separate artificial vaginas used for each collection to prevent cross-contamination. Ejaculates were immediately assessed for wave motion (scored 0–5), colour/consistency (scored 1–5) and volume. Concentration counts were performed using a haemocytometer (Neubauer Improved; Precicolor HBG, Giessen-Lutzellinden, Germany). All procedures were approved by the University of Sydney’s Animal Ethics Committee (permit number 2016/1106).

#### 2.1.2. Assessment of Sperm Kinematic Traits

The base media used for this study was kept as simple as possible (phosphate buffered saline (PBS) supplemented with 0.3% bovine serum albumin (BSA)) to avoid effects of nutrients in the media obscuring effects of seminal plasma supplementation. Samples were diluted to a standard concentration of 20 × 10^6^ spermatozoa mL^−1^ prior to measurement of sperm kinematic traits using computer assisted sperm analysis (CASA; Hamilton-Thorne IVOS II, Beverly, MA, USA). Sub-samples (5.5 µL) of diluted ejaculates were placed on pre-warmed (37 °C) CASA slides (Cell Vu; Millenium Sciences, Mulgrave, Vic., Australia), and 8 replicate videos (minimum of 200 cells) captured using factory ram settings (Animal Breeder software version 1.8). Parameters measured were: total motility, average path velocity (VAP), straight-line velocity (VSL), curvilinear velocity (VCL), linearity (LIN), straightness (STR), wobble (WOB), amplitude of lateral head displacement (ALH), and beat-cross frequency (BCF). Three replicate subsamples of each ejaculate at each timepoint were measured, and an average value calculated to minimise measurement error.

#### 2.1.3. Seminal Plasma Isolation

Ejaculates were centrifuged at 16,000× *g* (Model 1–14 Centrifuge; Sigma Aldrich) for 30 min at room temperature. The supernatant was collected and centrifuged for a further 30 min to remove remaining cell debris. Seminal plasma samples were then snap frozen and stored at −80 °C until the day of use.

### 2.2. Determining Seminal Plasma Concentration for Inter-Ejaculate Supplementation

A pilot study was conducted to determine at what concentration seminal plasma should be added to observe an effect. Freshly collected ejaculates (*n* = 3) were initially diluted to a standard 500 × 10^6^ spermatozoa mL^−1^ using our simple media (PBS + 0.3%BSA). Sub-samples were then further diluted to 20 × 10^6^ spermatozoa mL^−1^ with media including 0%, 5%, 12% or 20% seminal plasma (sourced from a pooled sample collected previously from rams housed at The University of Sydney Camden campus). Sperm kinematic traits of sub-samples were assessed using CASA immediately and 2 h after collection. The percentage of motile sperm increased in supplemented sub-samples across all concentrations, particularly after 2 h of exposure (Figure S1). Although there was a positive trend of increased change in motility with increased seminal plasma concentration, this was not significant (Table S1). There was a negative effect of seminal plasma concentration on change in sperm velocity, and a positive effect of time (Table S1, Figure S1). Hence, as supplementation of ejaculates with media containing 5% seminal plasma increased both sperm motility and velocity, this concentration was used in the main study.

### 2.3. Inter-Ejaculate Seminal Plasma Supplementation

#### 2.3.1. Donor Seminal Plasma Collection

Donor ejaculates were collected in June 2018, in four separate collections from each of the three rams. Sperm kinematic traits were measured using CASA, and then seminal plasma was isolated and frozen in 200 µL aliquots. Aliquots were thawed on ice as required for use in the main experiment.

#### 2.3.2. Ejaculate Level Effects of Seminal Plasma Supplementation

Four replicate blocks of the main experiment were completed in July 2018. Using a modified North Carolina II block design, in each replicate block, fresh ejaculates were collected from each of the three rams, and supplemented with seminal plasma from an ejaculate previously collected from each of the three rams. These supplemented subsamples were compared to an unsupplemented control subsample of each ejaculate (Table S2). This block design allowed us to test for interactive effects of the identity of the donor and receiver rams, as well as effects of individual characteristics of both the donor and receiver ejaculates. In the first block, a fresh ejaculate was not successfully collected from Ram C. Hence, the final sample size consisted of 11 focal ejaculates, each supplemented with seminal plasma from three different ejaculates. Sperm kinematic traits were measured using CASA at 0 and 2 h after collection.

### 2.4. Statistical Analysis

All statistical analyses were performed in R version 3.5, using lme4 and LmerTest packages. CASA measurements are highly correlated. Therefore, rather than analysing each trait separately, measures of ALH, BCF, LIN, STR, VAP, VCL, VSL, and WOB were combined using a Principle Component Analysis, and the dataset reduced to two axes which explained 86% of the total variation (Table S3). PC1, explaining 46% of variation, was interpreted as a measure of hyperactivity, as it was characterised by a positive loading of ALH and negative loadings of LIN, STR, WOB, VSL, BCF, and VAP (in decreasing order) (Table S3). PC2, explaining 40% of variation, was interpreted as a general measure of velocity, being characterised by positive loadings of VCL, VAP, VSL, and ALH (Table S3).

Ejaculate characteristics were assessed with linear models and t-tests. Ejaculate level effects were assessed with linear mixed effect models fit by REML. To test whether the relative quality of donor and receiver ejaculates was related to the outcome of supplementation, we examined the relationship between the difference in donor and receiver ejaculate traits versus the change in traits from control to supplemented subsamples. Ram (A, B, and C), donor (A, B, and C), and time (0 and 2 h) were also included as fixed factors, and date of trial included as a random factor. Interactions were assessed with log-likelihood ratio tests, and models reduced accordingly. Values are reported as mean ± s.e.

## 3. Results

### 3.1. Ejaculate Characteristics

**Figure 1:**
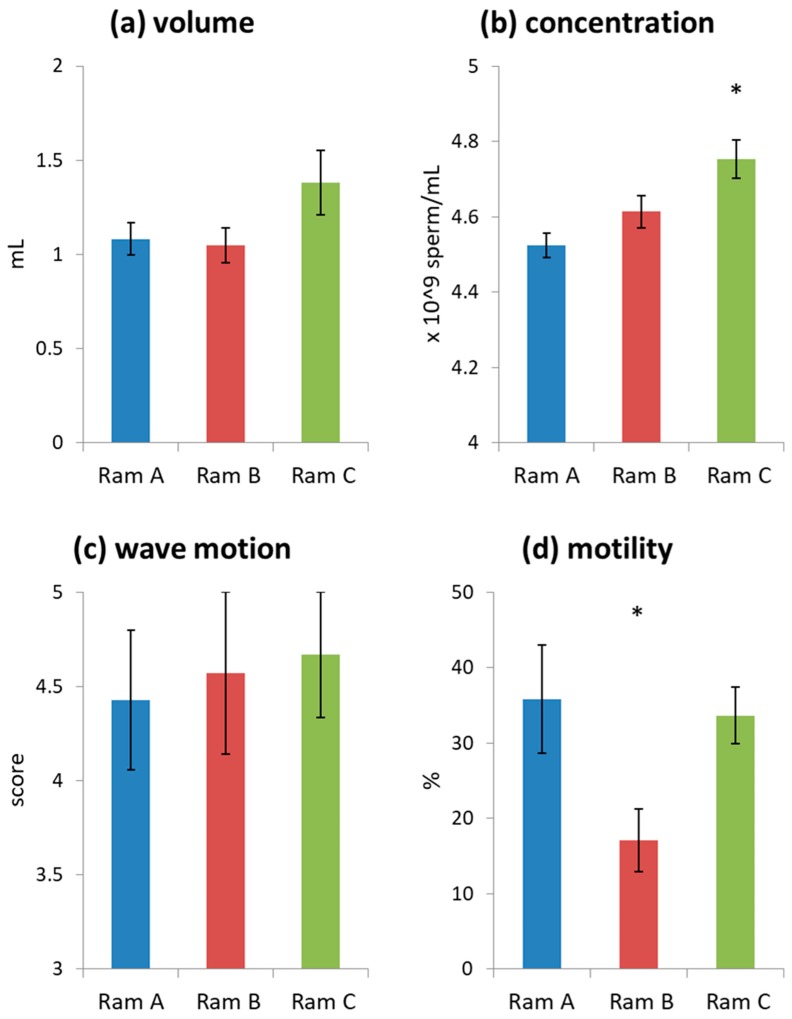
Average ejaculate characteristics grouped by Ram (mean ± s.e.): (**a**) undiluted ejaculate volume; (**b**) undiluted sperm concentration; (**c**) initial wave motion; (**d**) sperm motility of samples diluted with phosphate buffered saline (PBS) + bovine serum albumin (BSA) to a standard concentration of 20 × 10^6^ spermatozoa mL^−1^. Significant differences are marked with an *.

Initial assessment of all ejaculates used in the study indicated they were generally of high quality (volume = 1.16 ± 0.07 mL, wave motion = 4.55 ± 0.21, consistency = 5 for all ejaculates, concentration = 4.62 ± 0.03 × 10^9^/mL). Ram C produced ejaculates with a higher sperm concentration than Rams A and B (Figure 1, Table S4). Rams A and C produced ejaculates with higher sperm motility than Ram B (Figure 1, Table S4). Note that motility values are low as ejaculates were initially diluted with a simple media of phosphate buffered saline (PBS) supplemented with 0.3% bovine serum albumin (BSA) rather than a supportive sperm media. This was done as we wanted to see subtle effects of differences in seminal plasma supplementation which may have been obscured by the use of supportive media. Donor ejaculates tended to be slightly higher quality than receiver ejaculates (Figure S2), although these differences were not significant (Table S5). This was likely because the study was completed at the tail end of the breeding season and donor ejaculates were collected first.

### 3.2. Ejaculate Level Effects of Seminal Plasma Supplementation

**Figure 2:**
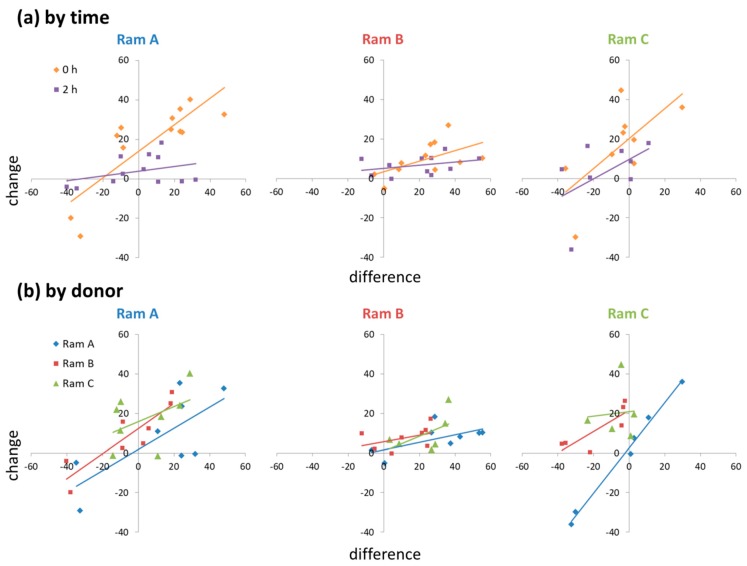
Ejaculate level effects of seminal plasma supplementation on changes in sperm motility: (**a**) RamxTimexDifference interactions; (**b**) RamxDonorxDifference interactions.

There was a positive relationship between the difference in motility between donor and receiver ejaculates and the change in motility from control to supplemented sub-samples (t = 5.620, *p* < 0.001). However, this relationship was context dependent, influenced significantly by both time point and ram ID (RamxTimexDifference: χ^2^ = 6.050, *p* = 0.049; RamxDonorxDifference: χ^2^ = 17.77, *p* = 0.001). The relationship was stronger at 0 h than at 2 h, and steeper in Rams A and C than Ram B (Figure 2). When acting as a donor, Ram A tended to have less of a positive effect on sperm motility than Rams B and C (Figure 2). When acting as a receiver, Ram B (who produced low motility ejaculates) showed little response in sperm motility to seminal plasma supplementation (Figure 2).

**Figure 3:**
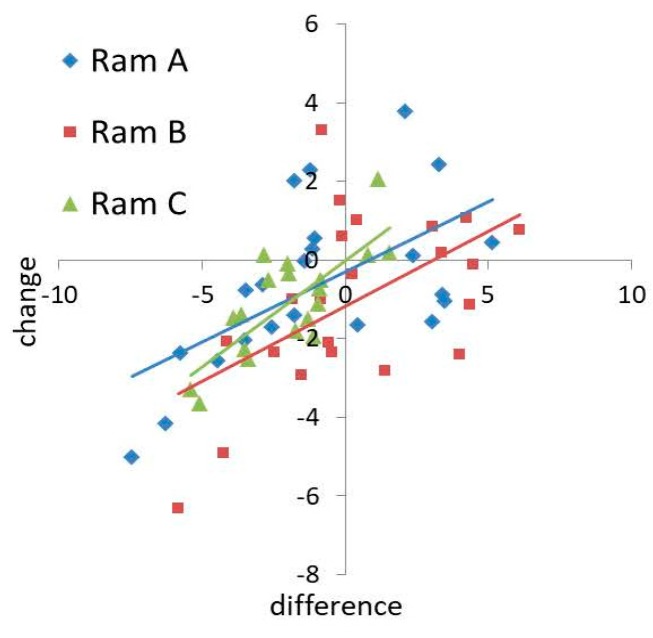
Ejaculate level effects of seminal plasma supplementation on changes in sperm hyperactivity (PC1).

There was also a positive relationship between the difference in hyperactivity (PC1) between donor and receiver ejaculates and change in hyperactivity with seminal plasma supplementation (t = 4.697, *p* < 0.001, Figure 3). No significant interactions were detected on the effects of predictor variables on hyperactivity (Full model versus Additive model: χ^2^ = 27.634, *p* = 0.538). Ram B had a greater negative effect on sperm hyperactivity than Rams A and C when acting as a donor (t = −2.370, *p* = 0.021, Figure 3). There was no significant relationship between the difference in velocity (PC2) and change in velocity (t = 1.397, *p* = 0.168). Receiver ejaculates of Ram C showed a larger change in velocity with supplementation than Rams A and B (t = 3.674, *p* = 0.001, Figure S3), and ejaculates of Ram C induced a greater change in velocity than Rams A and B when acting as a donor (t = 3.156, *p* = 0.003, Figure S3).

## 4. Discussion

Our study demonstrates that sperm motility is regulated (at least partially) at the ejaculate level by changes in seminal plasma composition, and that this variation can be utilised to adjust sperm traits in a predictable direction via inter-ejaculate supplementation. This effect is observed even when the seminal plasma is sourced from a previous ejaculate of the same male. However, the strength of the response depends on both the identity of the donor and receiver, indicating that some males may be suitable candidates to act as universal donors, and some males may fail to respond to seminal plasma supplementation. Our results also support the interpretation that instantaneous changes in sperm motility in response to cues of sperm competition are facilitated by changes in seminal plasma composition.

While our study provides a proof-of-principle, follow-up work is required to determine what changes in seminal plasma are driving the effects observed. It is possible that the relative proportion of fluids from each of the accessory glands are being altered, or specific molecular compounds within each of the fluids may be adaptively increased and/or decreased. While seminal plasma is a complex fluid composed of a multitude of organic and inorganic components [7,11], a promising place to begin investigations is the seminal proteome. While in principle, sperm motility may be increased simply by providing additional substrates (e.g., glucose) for energy metabolism, seminal fluid proteins are likely to modulate more complex effects on sperm function [20]. Protein is the largest component of seminal plasma (by weight), and is considered to be the dominant modulator of sperm function [16]. In addition, previous research examining effects of seminal plasma supplementation in rams has implicated proteins in modulating the effects of seminal plasma on spermatozoa freeze-thaw tolerance [17].

Similar to the directional effects observed in the current study, seminal plasma collected from rams with high freezing resilience was previously found to be able to significantly improve the post-thaw motility of rams with low freezing resilient spermatozoa, and conversely, a negative impact was seen from seminal plasma collected from rams with low freezing resilient seminal plasma [17]. When the composition of this phenotypically different seminal plasma was analysed, no significant differences were detected in terms of its ionic or biochemical analytes; however, it was determined that significant proteomic differences existed in terms of the abundance of certain seminal plasma proteins [17]. Several proteins were found to be correlated with high freezing resilience including 26S proteasome complex (PSMD13), heat shock protein 90 (HSP90), sorbitol dehydrogenase (SORD) and the TCP-1 complex (CCT), while others were found to be correlated to low freezing resilience such as zinc-2-alpha glycoprotein (AZGP1), angiogenin-2-like protein (ANG), cathepsin B (CTSB) and cystatin (CST3) [21]. Several of these proteins have also been linked to the variation seen in the liquid preservation of ram spermatozoa [22], which suggests a more immediate role or influence on sperm quality, similar to what was observed in the results of the current study. It could therefore be suggested that some of these proteins may be implicated in the changes in sperm kinetics observed.

One such protein, Zinc-2-alpha glycoprotein (AZGP1), is secreted by the prostate accessory sex gland and has been identified as an activator and intracellular modulator of human sperm motility [23]. AZGP1 has been shown to upregulate the cyclic AMP (cAMP)/cAMP-dependant protein kinase A (PKA) signalling pathway, which is known to be one of the significant signalling pathways necessary for the maintenance of mammalian spermatozoan motility [24]. The motility stimulating effect of this protein has been tested in ram spermatozoa and authors demonstrated that 1 ug/mL of recombinant AZGP1 significantly increased the motility and velocity of spermatozoa on the day of collection or at the time of supplementation. By 24 h of storage however, this stimulatory effect was detrimental to sperm preservation, with AZGP1 supplemented spermatozoa recording impaired motility compared to other treatments [22]. It is therefore plausible that the improvement in sperm kinetics observed in the above study, could be attributed to the presence of proteins such as AZGP1 in the seminal plasma of the donor rams. To test this theory the concentration of proteins including AZGP1, should be correlated with differences in sperm motility across multiple ejaculates from the same male, ideally while experimentally manipulating environmental conditions to determine what stimulus is driving ejaculate-level changes in seminal plasma composition.

Although future studies are required to uncover the molecular mechanisms underpinning effects observed in this study, it is unlikely that there is one simple change that explains inter-ejaculate variation in sperm motility. However, even if the mechanisms are yet to be determined, seminal plasma supplementation may be implemented to enhance motility during semen processing in ART immediately. Only a tiny amount of seminal plasma was required to elicit a change: 5% seminal plasma supplementation in the final dilution step was used in this study and in some cases resulted in a 40% increase in sperm motility. This means that the seminal plasma from a high quality ejaculate could be used to supplement many lower quality ejaculates. ART has been highly successful in increasing production efficiencies through the facilitation of selective breeding [25,26]. However, some males with desirable genetic traits may not be used as sires due to low fertility, meaning those beneficial genetic traits are lost from the breeding population. Seminal plasma supplementation may allow for the best of both worlds: seminal plasma from highly fecund males (even those without desirable production traits) may be used to boost the sperm function of low fertility males with desirable genetic traits. The regulation of sperm function by variation in seminal plasma composition means that as opposed to being considered a nuisance factor and something that needs to be controlled for, inter-ejaculate variability may be utilised to further increase the success rates of ART breeding programs.

## Supplementary Materials

The following are available online at https://www.mdpi.com/2079-7737/9/2/33/s1, Figure S1: Pilot study: Determining seminal plasma concentration for inter-ejaculate supplementation: (**a**) changes in sperm motility; (**b**) changes in average curvilinear velocity of sperm., Figure S2. Average ejaculate characteristics grouped by donor versus receiver (mean ± s.e.): (**a**) undiluted ejaculate volume; (**b**) undiluted sperm concentration; (**c**) initial wave motion; (**d**) sperm motility of samples diluted with PBS + BSA to a standard concentration of 20 × 10^6^ spermatozoa mL^−1^., Figure S3. Ejaculate level effects of seminal plasma supplementation on changes in sperm velocity (PC2): (**a**) change in sperm velocity of ejaculates of Rams A to C; (**b**) influence of seminal plasma from Rams A to C on the sperm velocity of receiver ejaculates., Table S1. Linear model output of pilot study data., Table S2. Experimental design, Table S3. Principal components analysis of sperm kinetic traits., Table S4. Linear model outputs of ejaculate characteristics grouped by Ram., Table S5. T-test results of ejaculate characteristics comparing donor to receiver traits.
