# Supplementary material for: From One Ejaculate to Another: Transference of Sperm Traits via Seminal Plasma Supplementation in the Ram
Source: Biology (Basel). 2020 Feb 18;9(2):33. doi: 10.3390/biology9020033 (PMC7168205; doi:10.3390/biology9020033)
Supplement: Supplementary file 1 [file biology-09-00033-s001.zip › biology-710011-supplementary.docx]

**Figure S1.** Pilot study: Determining seminal plasma concentration for inter-ejaculate supplementation: (**a**) changes in sperm motility; (**b**) changes in average curvilinear velocity of sperm.

**Figure S2.** Average ejaculate characteristics grouped by donor versus receiver (mean ± s.e.): (**a**) undiluted ejaculate volume; (**b**) undiluted sperm concentration; (**c**) initial wave motion; (**d**) sperm motility of samples diluted with PBS + BSA to a standard concentration of 20x10^6^ spermatozoa mL^-1^.

**Figure S3.** Ejaculate level effects of seminal plasma supplementation on changes in sperm velocity (PC2): (**a**) change in sperm velocity of ejaculates of Rams A to C; (**b**) influence of seminal plasma from Rams A to C on the sperm velocity of receiver ejaculates.

**Table S1.** Linear model output of pilot study data

|  | **% motility** | | **VAP** | | **VCL** | |
| --- | --- | --- | --- | --- | --- | --- |
|  | Co-ef | p | Co-ef | p | Co-ef | p |
| **Full model** | | | | | | |
| Concentration | 0.688 | 0.266 | -0.538 | 0.626 | 0.688 | 0.266 |
| Time | 13.369 | 0.267 | 42.699 | 0.059 | 13.370 | 0.267 |
| Conc × Time | -0.124 | 0.885 | -1.899 | 0.228 | -0.124 | 0.885 |
| **Reduced model** | | | | | | |
| Concentration | 0.626 | 0.144 | -1.488 | 0.072 | -2.456 | **0.022** |
| Time | 11.839 | **0.031** | 19.272 | 0.059 | 36.867 | **0.007** |

**Table S2.** Experimental design (A = ejaculate from Ram A, SP.A = seminal plasma from Ram A)

|  |  |  | **Donor** | | |
| --- | --- | --- | --- | --- | --- |
|  |  | **Control** | **A** | **B** | **C** |
| **Receiver** | **A** | A | A + SP.A | A + SP.B | A + SP.C |
|  | **B** | B | B + SP.A | B + SP.B | B + SP.C |
|  | **C** | C | C + SP.A | C + SP.B | C + SP.C |

**Table S3.** Principal components analysis of sperm kinetic traits.

|  | **PC1** | **PC2** |
| --- | --- | --- |
| Standard deviation | 1.927 | 1.785 |
| Proportion of variance | 0.464 | 0.398 |
| **Trait loadings** |  |  |
| ALH | 0.347 | 0.410 |
| BCF | -0.215 |  |
| LIN | -0.506 |  |
| STR | -0.496 |  |
| VAP | -0.186 | 0.518 |
| VCL |  | 0.556 |
| VSL | -0.246 | 0.489 |
| WOB | -0.484 |  |

**Table S4.** Linear model outputs of ejaculate characteristics grouped by Ram

|  | **Ram B** | | **Ram C** | |
| --- | --- | --- | --- | --- |
| **Response variable** | Co-ef | p | Co-ef | p |
| **Volume** | -0.033 | 0.836 | 0.297 | 0.096 |
| **Concentration** | 0.089 | 0.146 | **0.228** | **0.002** |
| **Wave motion** | 0.143 | 0.791 | 0.238 | 0.672 |
| **Motility** | **-18.776** | **0.022** | -2.178 | 0.782 |
| **VAP** | -5.128 | 0.709 | 7.884 | 0.583 |
| **VCL** | 0.603 | 0.977 | -1.910 | 0.931 |

**Table S5.** T-test results of ejaculate characteristics comparing donor to receiver traits

| **Response variable** | **t** | **p** |
| --- | --- | --- |
| **Volume** | 0.775 | 0.452 |
| **Concentration** | 0.064 | 0.949 |
| **Wave motion** | 0.315 | 0.756 |
| **Motility** | 1.627 | 0.119 |
| **VAP** | 0.862 | 0.400 |
| **VCL** | 0.651 | 0.525 |
